# Preparation and Application of In-Situ Loaded Silver Nanoparticles Antibacterial Fresh-Keeping Composite Paper

**DOI:** 10.3390/polym14183798

**Published:** 2022-09-11

**Authors:** Guangzhi Lin, Xia Li, Chuanshan Zhao

**Affiliations:** State Key Laboratory of Biobased Material and Green Papermaking, Faculty of Light Industry, Qilu University of Technology (Shandong Academy of Sciences), Jinan 250353, China

**Keywords:** in-situ load, silver nanoparticles, cellulose material, cherry, fresh-keeping, 1-MCP

## Abstract

The freshness and safety of fruits and vegetables affect our daily life. Paper products are often used in the packaging and transportation of fruits and vegetables, and these can provide other functions besides packaging after certain modifications and additions. In this study, the AgNPs/1-MCP antibacterial fresh-keeping composite paper was prepared by in-situ loaded silver nanoparticles and spraying 1-MCP solution. Moreover, the prepared paper was used to preserve sweet cherries. It was found that the prepared AgNPs/1-MCP antibacterial fresh-keeping composite paper could effectively inhibit *E. coli* and *S. aureus*. When the addition of 1-MCP in the paper was 0.05 g, the fresh-keeping effect on cherries was the best. Under this optimal condition, the weight loss ratio of the cherries was reduced by 1.93%, the firmness was increased by 27.7%, and the soluble solid content was increased by 25%. The preservation time was extended from 4 days to 12 days, three times that of the untreated ones. The prepared fresh-keeping material is environmentally friendly, non-toxic and harmless, simple to prepare and convenient to use, and is expected to become one of the important fresh-keeping methods for fruits.

## 1. Introduction

Paper has been widely used as a natural environmental protection packaging material. However, during the production, storage, and application of paper, it will be contaminated with bacteria and viruses as cellulose fiber provides good growth conditions for bacteria and viruses. Taking advantage of cellulose fibers’ environmentally friendly, degradable, and renewable characteristics, the antibacterial products were prepared in combination with antibacterial materials such as metal salts, halogen compounds, etc. [1,2,3,4,5,6]. Metal oxides such as ZnO and CuO are also widely used as antibacterial agents. Jamnongkan et al. [7] successfully prepared nonwoven fiber mats by combining the chemical stability and strong antibacterial properties of ZnO with PVA. The prepared nonwoven fiber mats are potentially attractive for applications such as wound dressings. However, the antibacterial properties of the antibacterial products will decline over time, and this may have the risks of introducing certain harms and toxicity to the human body.

Various broad-spectrum antimicrobials have successfully imparted antimicrobial activity to packaging over the past few decades, such as various essential oils (EOs) [8,9], chitosan [10], nisin [11,12] and more. In previous studies, metallic silver has demonstrated its antibacterial properties [13,14]. Compared with bulk-metal silver, silver nanoparticles have higher antibacterial properties and are ideal antibacterial materials [15,16]. Park et al. [17] combined cellulose fibers with metal nanoparticles through the covalent adsorption of silver nanoparticles and palladium nanoparticles. The results showed that the modified particles were well-dispersed in the fibers and highly antibacterial. Different from the antibacterial principle of other antibacterial agents, the antibacterial mechanism of silver is the interaction of silver ions with thiol groups in enzymes and proteins. These enzymes and proteins are a crucial part of bacterial survival, and the denaturation of bacterial proteins can play a role in inhibiting bacteria [18]. Silver is therefore inhibitory to most bacteria, has a long-lasting effect, rarely develops drug resistance, and shows negligible toxicity in humans.

Sweet cherries are rich in polyphenols, which have high antioxidant potential. Cherries have a short harvest time and a short market sales period. The main quality losses after harvest include moisture loss, softening, rot, and browning [19]. Extending sweet cherries’ fruit quality and shelf life are feasible through good handling and proper postharvest techniques without compromising eating quality. C_2_H_4_ (Ethylene) is a plant hormone, and a trace amount of C_2_H_4_ may accelerate the respiration of fruit, leading to rapid ripening, senescence, and softening of fruit [20]. The C_2_H_4_ binding to C_2_H_4_ receptors prompts the fruit to produce more C_2_H_4_, recycling the process of C_2_H_4_ binding to C_2_H_4_ receptors [21,22]. Therefore, using C_2_H_4_ inhibitors to prolong the storage time of fruits is now a common practice. 1-MCP is a synthetic compound in a gaseous state at standard temperature and pressure. It can specifically affect fruits at a shallow concentration and is environmentally friendly and non-toxic. The U.S. Environmental Protection Agency (EPA) approved this product for use in 1999 [23]. 1-MCP has been used in postharvest experiments on a variety of fruits and vegetables, including guavas [24,25], prunes [26], plums [27], and pears [28]. The most common use of 1-MCP is to treat fruit by fumigation to achieve the effect of preservation [29]. This method is cumbersome, and the fruit may also restore the level of C_2_H_4_ production after treatment, thereby starting the process of C_2_H_4_-induced fruit ripening again [30].

As far as is known, few studies combine cellulose paper-based materials, silver nanoparticles, and 1-MCP as fresh-keeping materials. Therefore, in this study, softwood fibers were used as substrates, and silver nanoparticles were loaded onto softwood fibers by in-situ loading and made into paper, then sprayed with 1-MCP to study the fresh-keeping effect of cherries in storage. A kind of fresh-keeping material with multiple functions, environmental friendliness, and that is non-toxic and harmless may provide new ideas for the field of fresh-keeping.

## 2. Materials and Methods

### 2.1. Materials and Reagents

The sweet cherries were selected from the Wufeng Mountain Cherry Plantation in Jinan, Shandong. After picking, they were quickly transported to the State Key Laboratory of Qilu University of Technology for sorting and selection. Similar fruits were selected according to size, shape, color, maturity, etc., and the damaged and defective fruits were removed.

Softwood pulp was purchased from Metsä Fibre Group (Espoo, Finland). Shellac was provided by Shandong Xuyang Chemical Import & Export Co., Ltd. (Heze, China). Poly-amide epichlorohydrin resin (PAE) was purchased from Shandong Tongchuang Chemical Co., Ltd. (Linyi, China). α-Cyclodextrin/1-MCP was provided by Xiqin Biotechnology Co., Ltd. (Xi’an, China). Plate Count Agar and LB broth were provided by Beijing Aoboxing Biotechnology Co., Ltd. (Beijing, China). Escherichia coli (*E. coli*) and Staphylococcus aureus (*S. aureus*) were purchased from Beijing Microbiological Culture Collection Center (Beijing, China).

### 2.2. Preparation of Silver Nanoparticle Paper

AgNO_3_ aqueous solutions with concentrations of 0.01, 0.05, and 0.1 mol/L were prepared at room temperature, and then NaBH_4_ aqueous solutions with different concentrations were prepared according to the molar ratios of AgNO_3_:NaBH_4_ of 1:1, 1:2, and 1:4. 2 g of 34 °SR softwood absolute dry pulp was weighed, AgNO_3_ aqueous solution was added, and the fibers were dispersed by stirring with a stirrer. Subsequently, suction filtration was performed with a circulating water vacuum pump (SHZ-D (III), Shanghai Dongxi Refrigeration Equipment Co., Ltd., Shanghai, China), and the fibers were washed with deionized water. They were then taken out and put in a NaBH_4_ solution for reaction. After the reaction, the free silver ions were repeatedly washed with deionized water and filtered by suction, which are the composite fibers with silver particles supported in situ. We took 2 g of silver nanoparticle-loaded composite fibers, added 1% PAE and converted the mixture into a wet web using a handsheet former (PK-3A, Austria PTI Co., Vienna, Austria). Both sides were covered with clean filter paper, vacuum dried for 10 min, and finally the water was equilibrated at room temperature to obtain silver nanoparticle paper. Equation (1) is the reaction equation [31].

(1) $${\mathrm{AgNO}}_{3}+{\mathrm{NaBH}}_{4}\to \mathrm{Ag}↓+\frac{1}{2}H_{2}↑+\frac{1}{2}B_{2}H_{6}↑+{\mathrm{NaNO}}_{3}$$

### 2.3. Preparation of AgNPs/1-MCP Antibacterial Fresh-Keeping Composite Paper

**Figure 1:**
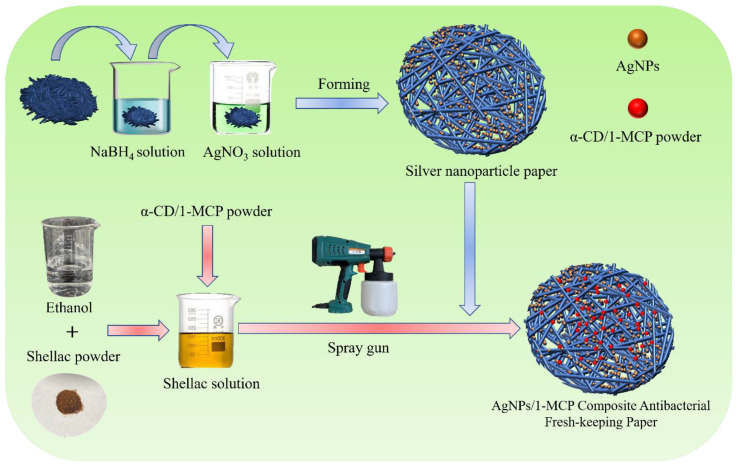
Preparation process of AgNPs/1-MCP antibacterial fresh-keeping composite paper.

According to previous research, shellac was added to absolute ethanol to form a 13 wt% solution, and a certain amount of α-cyclodextrin/1-MCP powder was added to it. After mixing evenly, the silver nanoparticle paper was sprayed with a hand-held electric spray gun (Ningbo Huipu Hardware Tools Co., Ltd., Ningbo, China), then dried at room temperature and stored in a ziplock bag. The preparation process of AgNPs/1-MCP antibacterial fresh-keeping composite paper is shown in Figure 1.

### 2.4. Characterization and Analysis of Antibacterial Fresh-Keeping Paper

The surface morphology of silver nanoparticle paper was characterized using scanning electron microscopy (SEM; Tescan Mira4, TESCAN ORSAY HOLDING, a.s., Brno-Kohoutovice, Czech Republic). Energy dispersive spectroscopy (EDS) elemental mapping images were obtained during SEM testing. The positions of the absorption peaks were detected by an ultraviolet spectrophotometer (UV-vis; SP-723PC, Shanghai Spectrum Instrument Co., Ltd., Shanghai, China). The fabricated silver nanoparticle paper was detected by an X-ray diffractometer (XRD; D8-ADVANCE, Bruker Co., Karlsruhe, Germany), and the goniometer was scanned stepwise from 20° to 90° in the 2*θ* range.

### 2.5. Testing of Tensile Index of Composite Paper

The prepared composite paper was subjected to a tensile test on a universal tensile machine (ZL-100A, Dalian Paper Testing Instrument Factory, Dalian, China). The composite paper was cut into a width of 15 mm, the tensile speed was set to 50 mm/min, 5 samples were measured for each sample, and the average value was taken to obtain the tensile strength. The tensile index was calculated from the test results by the formula, and the formula is as follows:

(2) $$Y=\frac{S}{g}\times 10^{3}$$

*Y* is the tensile index, N·m/g; S is the tensile strength, N/m; g is the basis weight of the paper (the basis weight is 60 g/m^3^), g/m^3^.

### 2.6. Antibacterial Effect of Antibacterial Fresh-Keeping Paper

The antibacterial activity of antibacterial fresh-keeping paper against Gram-negative bacteria *E. coli* and Gram-positive bacteria *S. aureus* was studied using the inhibition zone as follows:

The agar solution and broth were prepared in a 250 mL conical flask. The flask was then sealed, put into a fully automatic autoclave (CLG-32L, Tomy Digital Biology, Tokyo, Japan), sterilized at 121 °C for 20 min, and then taken out after cooling down to 50 °C. The ultra-clean workbench was irradiated with a UV lamp for 30 min and the operation took place in the ultra-clean workbench. We measured 20 mL of agar in a graduated cylinder and poured it into a petri dish. Some bacteria (*E. coli*, *S. aureus*) were inoculated in the broth and shaken in a constant temperature-shaking box (ZC-110B, Changzhou Zhongcheng Instrument Manufacturing Co., Ltd., Changzhou, China) at 37 °C and 150 pm for 12 h. The broth turned cloudy. A total of 70 μL of bacterial broth was dropped on the solidified agar, spread evenly with a spatula and placed on a paper sample. The samples were placed in a constant temperature and humidity incubator (ZC-800-1P, Qingdao Zhengchen Runke Testing Instrument Co., Ltd., Qingdao, China) at 37 °C for several hours to observe the size of the inhibition zone.

### 2.7. Preservation Experiment of Cherries

The AgNPs/1-MCP antibacterial fresh-keeping composite paper was placed in a polystyrene foam box, and 30 sifted sweet cherries were put in, covered with a layer of the same antibacterial fresh-keeping paper, and sealed. In the same operation, silver nanoparticle paper was placed as a control group without any treatment and only sprayed with Shellac solution.

#### 2.7.1. Weight Loss Ratio of Cherries

The formula for calculating the weight loss ratio is as follows:

$$Weight\;loss\;ratio\;\left(\%\right)=\frac{M_{n}}{M_{0}}\times 100\%$$

*M_n_* is the sum of the daily weight loss of sweet cherries after *n* days of storage, and *M*_0_ is the weight of the 0th day of storage.

#### 2.7.2. C_2_H_4_ (Ethylene) Concentration

A portable gas detector JK40-M3 (Shenzhen Jishunan Technology Co., Ltd., Shenzhen, China) was inserted into the hole reserved in the box to detect it on the 1st, 2nd, 3rd, 6th, 9th, 12th and 15th days. The unit is: mg/m^3^.

#### 2.7.3. Fruit Firmness of Cherries

After the thin peel was excised with a scalpel blade, the pulp firmness was measured individually on the sides and bottom of the equatorial circle of the selected cherries using the fruit firmness tester (FHT-05, Guangzhou Landtek Instrument Co., Ltd., Guangzhou, China). Penetration was measured using a 3.5 mm tip. The unit is: kgf/cm^2^.

#### 2.7.4. Fruit Firmness of Cherries

Three cherries were randomly selected from each group every three days. The pulp was taken and ground evenly, and the clear droplets were filtered off and placed on a hand-held refractometer (Tianjin Liaowang Photoelectric Technology Co., Ltd., Tianjin, China) to measure the total soluble solids (TSS) content, and obtain an average value, respectively.

#### 2.7.5. Bad-Fruit Ratio and Sensory Evaluation of Cherries

The bad-fruit ratio is one of the direct indicators for evaluating the fresh-keeping quality of the fruit. If there is apparent rot on the surface of the fruit, it is regarded as bad fruit. Generally, if the bad-fruit ratio exceeds 10%, it can be considered that this batch of fruits cannot be kept fresh. The ratio of bad fruit can be calculated by the ratio of the number of bad fruits to the total number of fruits.

$$Bad\;fruit\;ratio\;\left(\%\right)=\frac{B_{n}}{B_{0}}\times 100\%$$

*B_n_* is the sum of the daily number of bad fruits of sweet cherries stored for *n* days, and *B*_0_ is the total number of cherries.

The changes in the quality of sweet cherries were evaluated with various senses. The color, aroma, and fullness of the fruit pulp of sweet cherries were used as evaluation criteria, and each group of fruits was scored. Less than or equal to 6 points out of 10 points can be considered as out of shelf life. The bad-fruit ratio and sensory evaluation are both referred to for the preservation effect of cherries in this experiment. If one of them is lower than the value of the sales value of the commodity, it can be considered as having no sales value. The scoring criteria and corresponding scores are shown in Table 1.

## 3. Results and Discussion

### 3.1. Characterization of Silver Nanoparticle Paper

**Figure 2:**
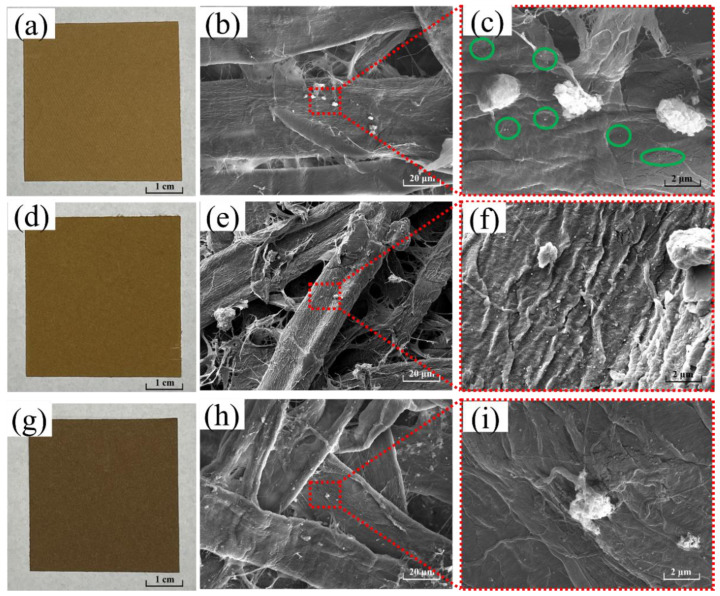
Photographs and SEM micrographs of silver nanoparticle paper. Molar ratio of AgNO_3_: NaBH_4_ = 1:4, AgNO_3_ concentrations were 0.01 mol/L (**a**–**c**), 0.05 mol/L (**d**–**f**), 0.1 mol/L (**g**–**i**).

The sprayed shellac will make the SEM unable to observe the silver nanoparticles, affecting the position of the UV-vis and XRD peaks. Therefore, the silver nanoparticle paper without shellac spraying was selected for detection. As we can see from the photograph (Figure 2a,d,g), with the increasing of AgNO_3_ concentration, the prepared paper turned out different colors, from brownish yellow (Figure 2a, 0.01 mol/L), to brown (Figure 2d, 0.05 mol/L), and brown-gray (Figure 2g, 0.1 mol/L). The results showed that silver particles do form in the paper. Increasing the concentration of NaBH_4_ has no obvious effect on the deepening of the paper color other than increasing the concentration of AgNO_3_. The reason is that the high concentration of NaBH_4_ will only make the silver nanoparticles disperse more evenly [32].

At the same AgNO_3_ concentration, the higher the NaBH_4_ concentration, the more uniform and stable the silver nanoparticles formed [32]. Therefore, the silver nanoparticle papers with different AgNO_3_ concentrations (AgNO_3_: NaBH_4_ = 1:4) were selected to character by SEM, although at low AgNO_3_ concentration (0.01 mol/L) (Figure 2b,c), uniform and stable silver nanoparticles can be formed. When the concentration of AgNO_3_ increased to 0.05 mol/L, a large number of uniform silver nanoparticles with nano-scale size was observed (Figure 2e,f). However, when the AgNO_3_ concentration wass up to 0.1 mol/L (Figure 2h,i), a large agglomeration of silver particles formed due to the high concentration of silver ions. Those silver particles lose their practical bacteriostatic effect during the time they are not nano-scale.

**Figure 3:**
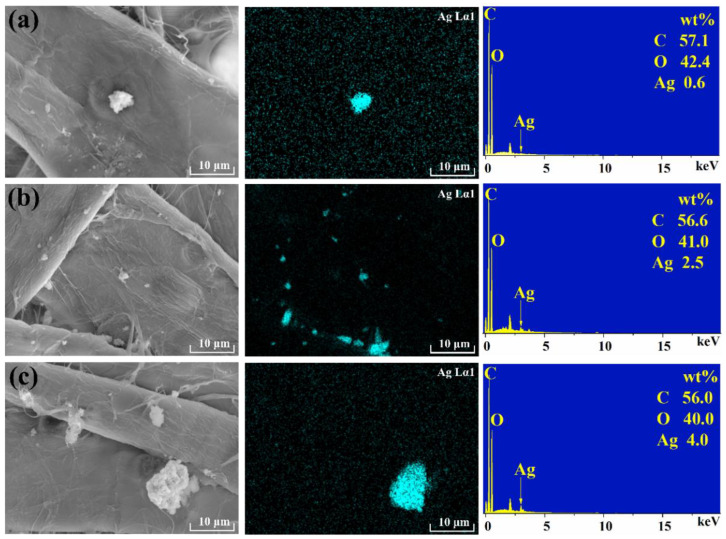
EDS of silver nanoparticle paper. Molar ratio of AgNO_3_: NaBH_4_ = 1:4, AgNO_3_ concentrations were 0.01 mol/L (**a**), 0.05 mol/L (**b**), 0.1 mol/L (**c**).

EDS showed that the prepared particles were Ag regardless of their size (Figure 3). The Ag content increases with the increase of AgNO_3_.

### 3.2. Tensile Index of Composite Paper

**Figure 4:**
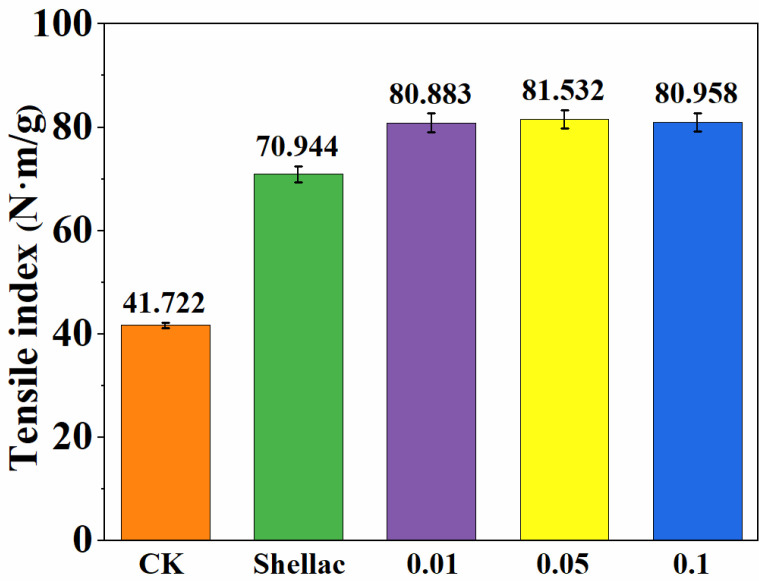
Tensile index of composite paper. Molar ratio of AgNO_3_:NaBH_4_ = 1:4, AgNO_3_ concentrations was 0.01, 0.05 and 0.1 mol/L.

As shown in Figure 4, the tensile index of the blank paper without silver nanoparticles was 41.722 N·m/g, while the tensile index was significantly improved after spraying with Shellac, reaching 70.944 N·m/g. After spraying with Shellac, the tensile index of silver nanoparticle paper reached about 81 N·m/g. The tensile index of silver nanoparticle paper prepared with different concentrations of AgNO_3_ (AgNO_3_:NaBH_4_ = 1:4) had no significant difference. The in-situ loaded silver nanoparticles can slightly increase the tensile index of the paper, and Shellac can significantly improve the tensile strength of the paper.

### 3.3. UV Absorption Spectroscopic Analysis of Silver Nanoparticle Paper

**Figure 5:**
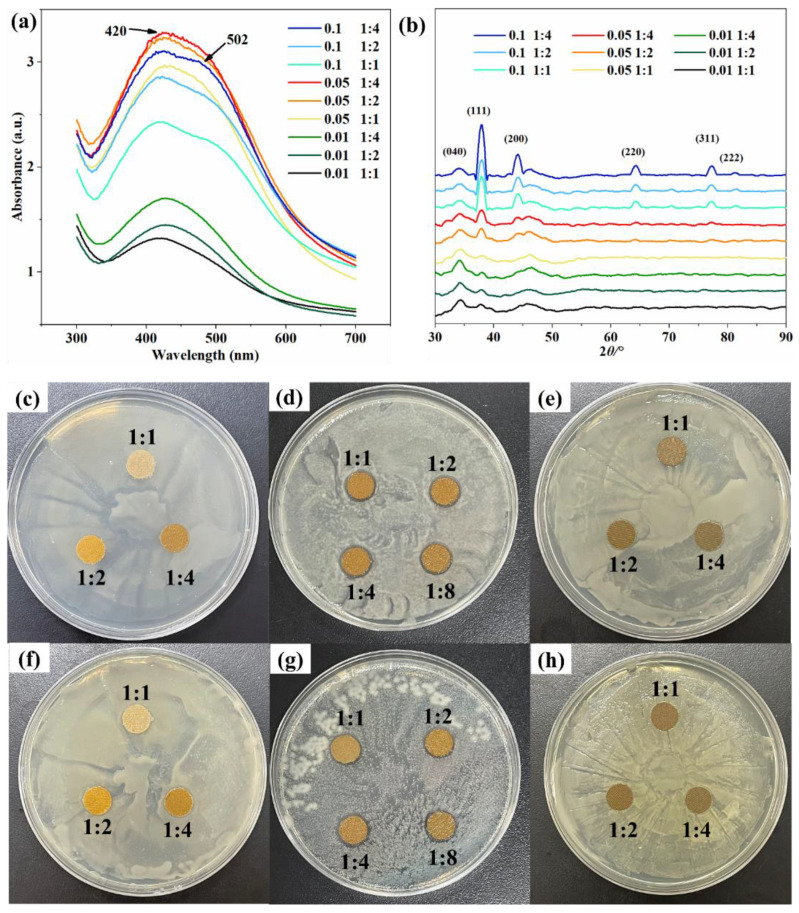
UV absorption spectra (**a**), XRD (**b**). The inhibition zone of *E. coli* (**c**–**e**) and *S. aureus* (**f**–**h**). AgNO_3_ concentrations were 0.01 mol/L (**c**,**f**), 0.05 mol/L (**d**,**g**), and 0.1 mol/L (**e**,**h**).

UV-vis spectroscopy is an effective method for particle characterization and can be used to characterize silver nanoparticles [33,34]. At different concentrations, the corresponding peaks of the prepared silver particles paper were around 420 nm (Figure 5a), indicating that silver particles were formed in the paper. The observed redshift and broadening of the absorption band were due to the increase in particle size and size distribution [35]. At a certain concentration of AgNO_3_, with the increasing of the NaBH_4_ concentration, the absorption peak displayed by the paper sample became higher. In other words, the absorption peak is constantly narrowing, indicating that the lower the molar ratio of AgNO_3_:NaBH_4_, the more stable and uniform the particle size of the silver nanoparticles formed. What is more, with the increasing of AgNO_3_ concentration, the silver particle size also increased gradually. At low dosage of AgNO_3_ (0.01 mol/L), the peaks formed were lower. Although relatively uniform silver particles of similar size were formed, the number was too small. At the 0.1 mol/L AgNO_3_ concentration, the absorption peak became broad, and an apparent shoulder appeared at 502 nm. It showed that at high AgNO_3_ concentration, the formed silver nanoparticles were not only spherical or quasi-spherical but also other different shapes [36]. However, when the concentration of AgNO_3_ was 0.05 mol/L, no other impurity peaks appeared, therefore 0.05 mol/L is a more suitable concentration.

### 3.4. X-ray Diffraction Analysis of Silver Nanoparticle Paper

The XRD patterns at different concentrations showed a characteristic peak at 34.16° (Figure 5b), corresponding to the (040) crystal plane of type I cellulose fibers [37,38,39]. In addition, there were five peaks located at 37.9°, 44.14°, 64.28°, 77.22° and 81.22°, corresponding to the (111), (200), (220), (311) and (222) crystal planes of silver [40]. The (311) and (222) are missing at low concentrations (AgNO_3_ concentration is 0.01 mol/L). The analysis may be because the number of silver nanoparticles formed was too small, and the crystal plane strength is insufficient, which is not reflected in the XRD pattern. It can be judged that no other impurities were introduced into the prepared silver nanoparticle paper.

### 3.5. Antibacterial Effect of AgNPs/1-MCP Antibacterial Fresh-Keeping Composite Paper

AgNPs/1-MCP antibacterial fresh-keeping composite paper properties against *E. coli* (Figure 5c–e) and *S. aureus* (Figure 5f–h) were determined by zone of inhibition.

When the AgNO_3_ concentration was 0.01 mol/L, there was no significant inhibition zone (Figure 5c,f). The reason may be that the AgNO_3_ concentration was not high enough, resulting in too few silver nanoparticles loaded on the fibers, so that there was no strong bacteriostatic effect. The paper samples with an AgNO_3_ concentration of 0.1 mol/L also had no antibacterial effect (Figure 5e,h). It may be that the concentration of AgNO_3_ was too high, and the reduced silver agglomerates formed large silver particles, thus losing the high antibacterial effect of silver nanoparticles. The silver nanoparticle paper with an AgNO_3_ concentration of 0.05 mol/L has a significant antibacterial effect (Figure 4d,g). With an increasing of NaBH_4_ concentration, the size of the inhibition zone tended to be stable. When the molar ratio of AgNO_3_:NaBH_4_ was increased from 1:4 to 1:8, the bacteriostatic effect hardly improved. Therefore, at optimized condition (0.05 mol/L, 1:4), the paper showed the best comprehensive effect.

### 3.6. Preservation Experiment of Cherries

When the dosage of AgNO_3_ is 0.05 mol/L and NaBH_4_ is 0.2 mol/L, the prepared silver nanoparticle antibacterial paper has a relatively uniform particle size distribution, sufficient silver nanoparticles, and the best antibacterial effect. In order to explore the effect of 1-MCP on the preservation effect, the preservation of cherries at different concentrations of 1-MCP was studied. The weight loss ratio, firmness, C_2_H_4_ concentration, TSS, bad fruit ratio and sensory qualities of cherries were analyzed and evaluated. CK is the blank control group without spraying, Shellac is the control group with spraying shellac, 0.001 is the group with 0.001 g of 1-MCP added, 0.005 is the group with 0.005 g of 1-MCP added, and so on.

#### 3.6.1. Weight Loss Ratio of Cherries

**Figure 6:**
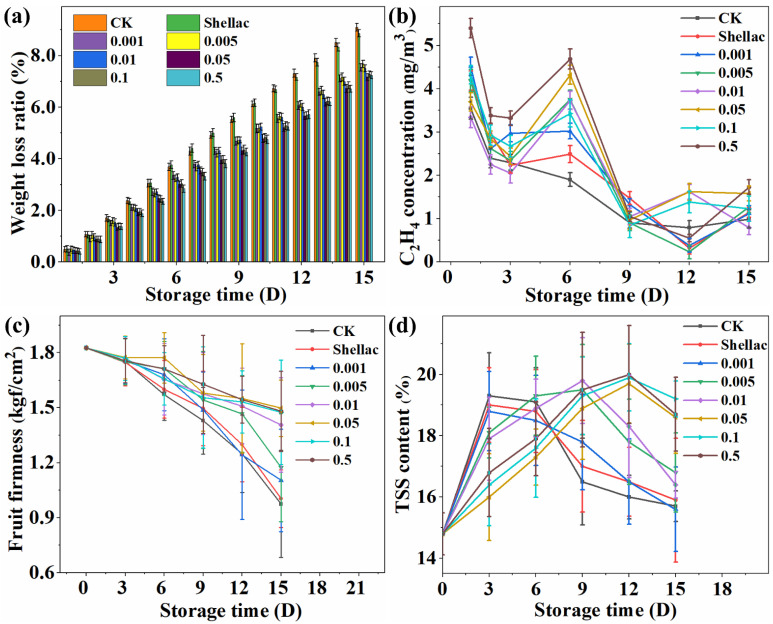
Weight loss ratio (**a**), C_2_H_4_ concentration (**b**), Fruit firmness (**c**), and total soluble solids content (**d**).

After cherries are picked, they will still carry out physiological activities such as respiration and metabolism [41], which will cause the fruit to consume its water and nutrients, resulting in weight loss [42]. Therefore, it is of great significance to study the change in the weight loss ratio during the preservation of cherries, which is of great significance to the changes in the freshness of cherries and the strength of the preservation effect. Changes in the weight of the different experimental groups during 15 days were determined every day during 15 days of storage (Figure 6a). With time going on, the weight loss ratio of CK and the Shellac groups (without 1-MCP) was similar, and higher than others, indicating that Shellac had no preservation effect. Compared with the CK group, the weight loss ratio of the three groups with the addition of 0.001 g, 0.005 g, and 0.01 g of 1-MCP was significantly lower, indicating that the lower addition of 1-MCP could have an apparent fresh-keeping effect. Compared with effects and additional amounts, it can be concluded that the optimal addition amount of 1-MCP is 0.05 g. The weight loss ratio of the 0.05 g experimental group was 1.93% lower than that of the control group.

#### 3.6.2. C_2_H_4_ (Ethylene) Concentration

The overall trend of C_2_H_4_ concentration was similar for all groups (Figure 6b). At high dosage of 1-MCP (0.05 g, 0.1 g, 0.5 g), C_2_H_4_ concentration was higher. With decreasing dosage of 1-MCP, the C_2_H_4_ concentration did not significantly lower. At low dosage of 1-MCP (0.001 g, 0.005 g, 0.01 g), the C_2_H_4_ concentration was mostly lower than that of the high addition group. In others, the C_2_H_4_ concentration of these two groups was lower than that of the experimental group dosed with 1-MCP. The reason for the analysis may be that the CK group and Shellac group did not add 1-MCP, and the C_2_H_4_ produced by cherries, in turn, promoted the ripening of cherries. C_2_H_4_ is involved in the process of production, consumption, and regeneration. After adding 1-MCP, 1-MCP binds to the C_2_H_4_ receptor so that C_2_H_4_ cannot be combined with the C_2_H_4_ receptor, and the cycle of generation, consumption and regeneration cannot continue. Therefore, the C_2_H_4_ concentration of the experimental group with 1-MCP addition was higher than that of the CK and Shellac groups.

#### 3.6.3. Fruit Firmness of Cherries

Fruit firmness is one of the essential indicators to measure fruit maturity and storage quality [43]. In the cherry ripening and ageing process, the pectin in the cherry pulp gradually dissolves [44], and the firmness gradually decreases [45]. By measuring the cherry pulp’s firmness, the cherry’s degree of ripeness or softening can be determined, thereby judging the change in the quality of the cherry and the change in the storage time. The longer the storage time went on, the fruit firmness of cherries in the CK and Shellac groups decreased significantly (Figure 6c). Compared with the CK group, the effect of 1-MCP addition of 0.001 g and 0.005 g to maintain the firmness was slightly better, and 0.01 g was slightly lower than that of 0.05 g, 0.1 g, and 0.5 g. To sum up, in the control group that maintained the best firmness, the addition of 1-MCP of 0.05 g can maintain a higher level of freshness. The fruit firmness of the 0.05 g experimental group was 27.7% higher than that of the CK group.

#### 3.6.4. Total Soluble Solid Content of Cherries

The content of TSS (mainly soluble sugar) in cherries can directly reflect the ripeness and flavor changes of the fruit. For most sweet fruits, during the process of unripe–ripe–senescent, the TSS content showed a rising trend, reaching the peak and then decreasing. The continuous consumption of TSS in the storage of cherries leads to a decline in the quality of the cherries, resulting in a shortened shelf life [46]. As the storage time went on, the TSS content of all samples increased first and then decreased, which was in line with the natural physiological maturity to decay of cherries (Figure 6d). From Figure 5d, the TSS content of the CK group, the Shellac group, and the 0.001 g group reached the highest value at the fourth day and began to decline. When the addition of 1-MCP increased to 0.005 g or 0.01 g, the highest point of TSS content was at about the 7–8th day. Furthermore, the highest point of TSS content exceeded 12 days when the addition of 1-MCP reached 0.05 g. Compared with the CK group, the experimental group with a high 1-MCP addition amount (0.05 g, 0.1 g, 0.5 g) prolonged the preservation time by three times. This showed that adding 1-MCP can significantly prolong the fresh-keeping effect.

#### 3.6.5. Bad-Fruit Ratio and Sensory Evaluation of Cherries

**Figure 7:**
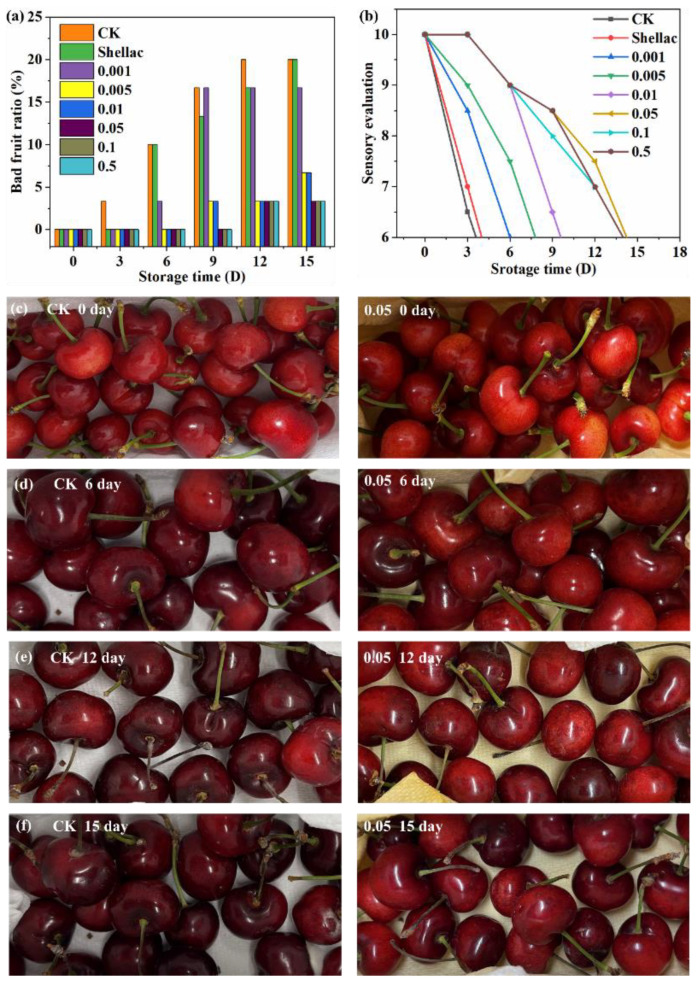
Bad-fruit ratio (**a**), sensory evaluation (**b**), comparison of the fresh-keeping effect of cherries with different 1-MCP additions (**c**–**f**), left 0 g, right 0.05 g.

During storage, cherries undergo a process of underripe, fully ripe, wilted, and rotten. When the bad-fruit ratio exceeds a certain level, it will reduce consumers’ desire to buy and its selling value. The bad-fruit ratio of the CK and the Shellac group reached 10% on the sixth day, which was no longer available for sale (Figure 7a). On the ninth day, the 0.001 g experimental group reached 15%. Continuously extending the storage time, the last five groups (0.005 g, 0.01 g, 0.05 g, 0.1 g, 0.5 g) did not have more than a 10% bad-fruit ratio. The reason may be that the moisture and nutrients of the cherries are continuously reduced, and they will only become shriveled.

The CK and Shellac group will be kept fresh for more than 3 days, and the sensory evaluation will decrease sharply and lose their selling value (sensory evaluation < 6) (Figure 7b, Table 2). When the sensory evaluation was lower than 6 points, 0.001 g, 0.005 g and 0.01 g took 6, 8 and 10 days, respectively, while the 1-MCP high-addition group (0.05 g, 0.1 g, 0.5 g) took 13–14 days. With the increasing addition of 1-MCP, the experimental group with higher 1-MCP content took a longer time for the sensory evaluation to be less than 6 points.

Figure 7c–f is the comparison chart of the storage time from 0 to 15 days between the CK group and the experimental group with 0.05 g of 1-MCP added. On the sixth day (Figure 7d), the CK group had turned black and had no fragrance, while the 0.05 g group was bright red with a slightly dark red color and still had a good fragrance and appearance. After 15 days of storage (Figure 7f), the color of the cherries in the 0.05 g group was dark red and still had the value for sale, while the CK group had already turned black and wilted. From the appearance, aroma, and fullness of the pulp, it can also be confirmed that the preservation time of the 0.05 g group was three times that of the CK group, and the storage time was extended from 4–5 days to 15 days.

### 3.7. Summary of Cherry Preservation Experiments

The following conclusions can be drawn by combining the weight-loss ratio, fruit firmness, TSS content, bad-fruit ratio, and sensory evaluation. The 1-MCP low-addition group (0.001 g, 0.005 g, 0.01 g) had a significantly longer preservation time than the untreated group (CK, Shellac), and the high-addition group (0.05 g, 0.1 g, 0.5 g) had a longer preservation time. When the addition of 1-MCP reached 0.05 g, the preservation effect reached its best, and the preservation experiment was extended from 4–5 days to 15 days.

**Figure 8:**
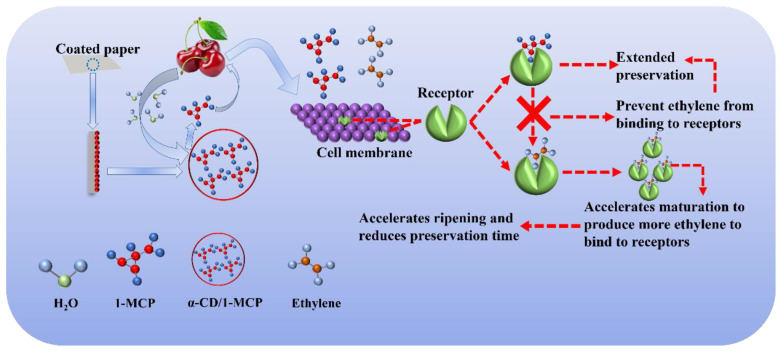
Schematic diagram of preservation of 1-MCP.

With the dissolving of α-CD in the water generated by the fruit’s respiration, 1-MCP was released from the coating layer (Figure 8). The primary role of 1-MCP is to bind to the C_2_H_4_ receptors in the fruit, thereby preventing the C_2_H_4_ receptors of the fruit from binding to C_2_H_4_, reducing the respiration, slowing down the aging speed of the fruit and prolonging the preservation time [47,48,49]. If the C_2_H_4_ receptors in fruits are likened to a “lock”, then C_2_H_4_ is the “true key”, which can activate the C_2_H_4_ receptors and trigger a series of chain reactions. The 1-MCP is the “fake key”, which can be inserted into the “lock” instead of the “real key” but cannot unlock the subsequent reaction, and the only function is to prevent the “real key” from entering the “lock” [50].

## 4. Conclusions

In this study, a green, non-toxic, low-cost, and degradable antibacterial and fresh-keeping material was prepared by a combination of in-situ loading, wet forming, and spraying. When the concentration of AgNO_3_ solution was 0.05 mol/L and the NaBH_4_ solution was 0.2 mol/L, the prepared silver nanoparticle paper had the best bacterial inhibition effect. When the amount of 1-MCP added was 0.05 g, the preservation effect on cherries was the best, and the preservation time was extended from 4 to 5 days in the CK group to 15 days. It can effectively reduce firmness and consumption of soluble solids of cherries during storage. The nutrient composition and flavor of cherries are maintained, and the bad-fruit and weight loss ratio of cherries is suppressed. All in all, the prepared antibacterial fresh-keeping paper has a solid temporary fresh-keeping function and is expected to be used as a material for fresh-keeping packaging in small and medium-sized planting areas and households.

## Figures and Tables

**Table 1:** Sensory Quality Evaluation Criteria.

Fullness of Fruit		Color		Fragrance	
Senses	Score	Senses	Score	Senses	Score
Full flesh	4	Bright red	2.5–3.0	Strong fragrance	2.5–3.0
Slightly soft	3	Red to dark red	2.0–2.5	Normal fragrance	2.0–2.5
Soft	2	Dark red	1.5–2.0	Fragrance fades	1.5–2.0
Shrivel	1	Black	0–1.5	No fragrance	0–1.5

**Table 2:** Cherry sensory evaluation results.

	Sample Name	CK	Shellac	0.001	0.005	0.01	0.05	0.1	0.5
Storage Time/Day									
0		10	10	10	10	10	10	10	10
3		6.5	7	8.5	9	10	10	10	10
6		4	4	6	7.5	9	9	9	9
9		2	3	3	5	6.5	8.5	8	8.5
12		1	1	3	4	4	7.5	7	7
15		1	1	2	3	3	5.5	5.5	5

Green: Has sale value. Yellow: Nearly no sale value. Red: not available for sale.

## Data Availability

The data presented in this study are available on request from the author.
